# 1533. The First Ten Years of HIV Preexposure Prophylaxis Use and Missed Opportunities at an Urban Academic Children’s Hospital

**DOI:** 10.1093/ofid/ofad500.1368

**Published:** 2023-11-27

**Authors:** Elizabeth Christian, Sanya Thomas, Lakshmi Ganapathi, Douglas Krakower

**Affiliations:** Boston Children's Hospital, Boston, Massachusetts; Boston Children's Hospital/Harvard Medical School, Boston, Massachusetts; Massachusetts General Hospital, Boston, Massachusetts; Harvard Medical School, Boston, MA

## Abstract

**Background:**

HIV preexposure prophylaxis (PrEP) is underprescribed in adolescents, with ∼16% of PrEP-eligible youth receiving prescriptions in 2019. We analyzed trends in PrEP prescribing and quality of care in adolescents at the largest academic pediatric hospital in New England to inform strategies to improve HIV prevention in this group.

**Methods:**

We reviewed charts of all patients prescribed PrEP at Boston Children’s Hospital between July 2012-December 2021 and extracted demographics, lab results and details of PrEP discussions. We extracted data on all patients with new HIV diagnoses to identify missed opportunities for PrEP.

**Results:**

From 2015-2021, 54 patients were prescribed PrEP; none were during 2012-2014. Prescriptions increased annually except for 2020, coinciding with the COVID-19 pandemic. Most PrEP patients (87%) were >18 years old and prescribing for those < 18 years old remained rare despite regulatory approval for this age-range in 2018 (Fig. 1).

Of the 54 PrEP patients,16% never initiated, 28% discontinued within 6 months, 31% had multiple short (median 3.5 months) periods of use, and 13% started once and continued for >6 months (median 12 months, range 7-36 months). (Fig. 2) The most common reason for discontinuing PrEP was loss to follow up (Fig. 3).

Patients initiated PrEP discussions in 78% of cases, and half of PrEP prescriptions were same day starts. Most patients accessed PrEP through adolescent (81%) or gender speciality (11%) clinics with only 4% through General Pediatrics. Nearly all patients (98%) had baseline HIV and Hepatitis B testing; of 41 patients using PrEP for >3 months, all had follow up HIV tests within 4 months.

Of 22 patients newly diagnosed with HIV, none had taken PrEP. The majority of HIV diagnoses were made during routine screening (68%); three were diagnosed at pre-PrEP screening. Most diagnoses (68%) were made by generalists.

**Figure 1:**
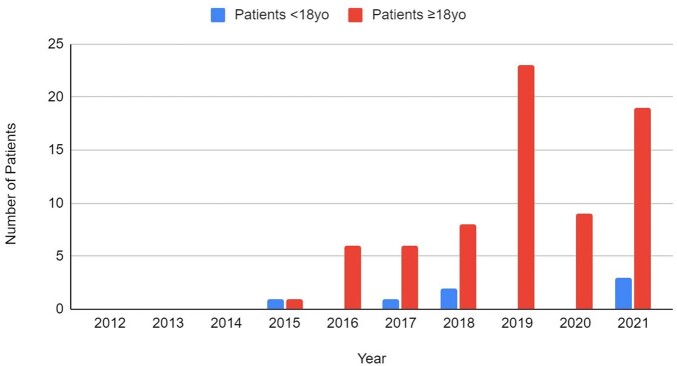
Number of Patients Prescribed PrEP per Year

**Figure 2:**
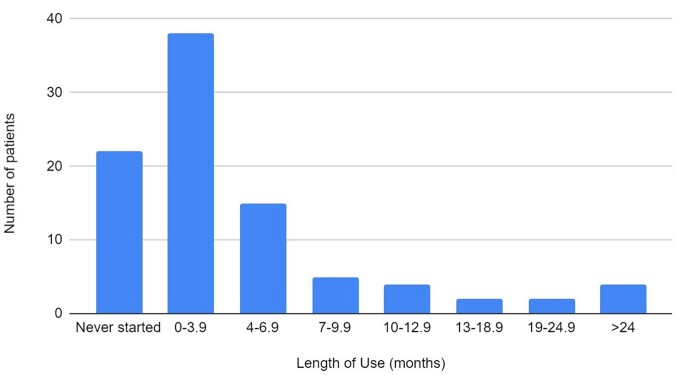
Duration of PrEP Use

**Figure 3:**
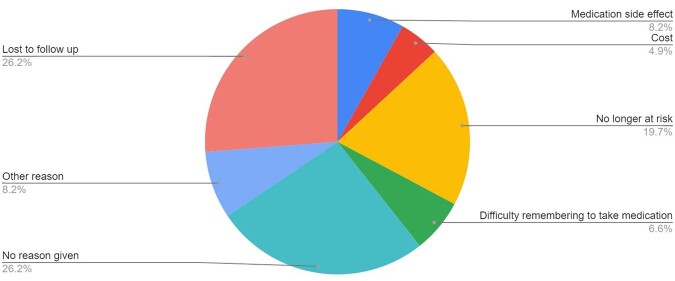
Reasons for PrEP Discontinuation

**Conclusion:**

Only 54 patients were prescribed PrEP during the past decade at a large pediatric hospital despite 22 new HIV diagnoses in this period. Non-initiations or discontinuations were common, suggesting a need for strategies to improve PrEP provision and persistence. With youth initiating most PrEP discussions, strategies to engage providers in PrEP are needed, in particular among generalists where most new HIV diagnoses occurred.

**Disclosures:**

**Douglas Krakower, MD**, Gilead: Grant/Research Support|Merck: Grant/Research Support|U. North Texas Health Sciences Center: Funding for mentoring|UAB: Advisor/Consultant|UpToDate, Inc.: Royalties|Virology Education: Honoraria

